# Increased Endothelial Cell-Leukocyte Interaction in Murine Schistosomiasis: Possible Priming of Endothelial Cells by the Disease

**DOI:** 10.1371/journal.pone.0023547

**Published:** 2011-08-10

**Authors:** Suellen D. S. Oliveira, Luis E. M. Quintas, Luciana S. Amaral, François Noël, Sandra H. Farsky, Claudia L. M. Silva

**Affiliations:** 1 Laboratory of Molecular and Biochemical Pharmacology, Biomedical Sciences Institute, Federal Universidade of Rio de Janeiro, Rio de Janeiro, Brazil; 2 School of Pharmaceutical Sciences, São Paulo University, São Paulo, Brazil; University of Illinois at Chicago, United States of America

## Abstract

**Background and Aims:**

Schistosomiasis is an intravascular parasitic disease associated with inflammation. Endothelial cells control leukocyte transmigration and vascular permeability being modulated by pro-inflammatory mediators. Recent data have shown that endothelial cells primed *in vivo* in the course of a disease keep the information in culture. Herein, we evaluated the impact of schistosomiasis on endothelial cell-regulated events *in vivo* and *in vitro*.

**Methodology and Principal Findings:**

The experimental groups consisted of *Schistosoma mansoni*-infected and age-matched control mice. *In vivo* infection caused a marked influx of leukocytes and an increased protein leakage in the peritoneal cavity, characterizing an inflamed vascular and cellular profile. *In vitro* leukocyte-mesenteric endothelial cell adhesion was higher in cultured cells from infected mice as compared to controls, either in the basal condition or after treatment with the pro-inflammatory cytokine tumor necrosis factor (TNF). Nitric oxide (NO) donation reduced leukocyte adhesion to endothelial cells from control and infected groups; however, in the later group the effect was more pronounced, probably due to a reduced NO production. Inhibition of control endothelial NO synthase (eNOS) increased leukocyte adhesion to a level similar to the one observed in the infected group. Besides, the adhesion of control leukocytes to endothelial cells from infected animals is similar to the result of infected animals, confirming that schistosomiasis alters endothelial cells function. Furthermore, NO production as well as the expression of eNOS were reduced in cultured endothelial cells from infected animals. On the other hand, the expression of its repressor protein, namely caveolin-1, was similar in both control and infected groups.

**Conclusion/Significance:**

Schistosomiasis increases vascular permeability and endothelial cell-leukocyte interaction *in vivo* and *in vitro*. These effects are partially explained by a reduced eNOS expression. In addition, our data show that the disease primes endothelial cells *in vivo,* which keep the acquired phenotype in culture.

## Introduction

*Schistosoma mansoni* causes an intravascular parasitic disease characterized by chronic, systemic inflammation. Adult schistosomes reside in the mesenteric portal venous system where they release antigens and female worms lay eggs, making endothelial cells a target of the disease with phenotypic changes reflecting the disease-mediated host immune modulation [1]–[4].

In the murine model and in human disease two polarized cytokine profiles are observed after infection. There is an early type-1 cytokine response characterized by the production of interferon-γ (INF-γ), interleukin (IL)-2 and tumor necrosis factor (TNF), evolving to a type-2 response due to the presence of eggs, and characterized mainly by IL-4, IL-5, IL-6 and IL-13 secretion [5]. The balance between the effects of these pro- and anti-inflammatory cytokines determines the outcome of the disease [5], [6].

Endothelial cells are important to vascular homeostasis since they regulate inflammatory events, such as vascular permeability, leukocyte rolling and adhesion to endothelial cell within microcirculation [7]–[10]. Additionally, endothelial cells are target of cytokines that alter cell function during inflammation [10], [11]. For instance, in schistosomiasis there is an increase in the plasma concentration of soluble intercellular adhesion molecule (ICAM)-1, a classical marker of endothelial activation in inflammation, which correlates to the severity of the disease [12]. Another characteristic of endothelial cells is that some phenotypic changes primed *in vivo*, such as the expression of adhesion molecules for instance, are kept *in vitro* [13], [14].

Although schistosomiasis is related to an inflammatory condition, and the major cytokines are well known, the knowledge about endothelial cell-leukocyte interaction in schistosomiasis is limited. Therefore, present work aimed to examine the influence of murine schistosomiasis on some endothelium-related events such as leukocyte adhesion, migration and vascular permeability, and also the influence of the disease on the expression of the constitutive endothelial nitric oxide synthase (eNOS; EC 1.14.13.39), whose product (nitric oxide, NO) inhibits leukocyte traffic and vascular permeability [15]–[17]. Our data suggest that murine schistosomiasis enhances vascular permeability and endothelial cell-leukocyte interactions *in vivo* and *in vitro*. These alterations relate, at least partially, to an endothelial cell phenotypic alteration characterized by decreased expression of eNOS and consequently its product NO, which are characteristics of endothelial dysfunction. In addition, our data strongly suggest that the disease primes murine endothelial cells *in vivo* for an increased leukocyte adhesion, which keep the information in culture.

## Methods

### Ethics statements

Male Swiss mice (75 to 90 days old) were used in all procedures following institutional guidelines for animal experiments (protocol DFBC-ICB-011). Animals were kept under a light/dark cycle of 12/12 h and had access to water and food *ad libitum*. All efforts were done to reduce the number of animals needed and to prevent animal suffering.

### Infection with Schistosoma mansoni

Animals were infected as previously described [18], [19]. Briefly, mice (7 days old) were exposed to 80 cercariae of both genders (BH strain; obtained from infected snails of *Biomphalaria glabrata* species) during 8 min for percutaneous infection. Animals were used 65–80 days after the infection in order to allow the full establishment of the infection.

### Leukocyte transmigration into peritoneal cavity and vascular permeability

The experimental groups consisted of control and *S. mansoni*-infected mice (body weight 28.0±1.5 g). For leukocyte transmigration assays, both groups were injected i.p. with vehicle (sterile phosphate buffered saline, PBS: NaCl 137 mM, Na_2_HPO_4_ 8.1 mM, NaH_2_PO_4_ 1.5 mM and KCl 2.7 mM, pH 7.4.). For vascular permeability assays, both control and infected animals were injected with 100 µl 1% Evans blue solution diluted in sterile PBS (i.v.).

Three hours after vehicle injection or 1 h after Evans blue solution injection, the anaesthetized animals were sacrificed followed by the intraperitoneal injection of 5 ml of sterile PBS. Subsequently the abdomen was massaged, the peritoneal exudate was collected (approximately 95% of the initial volume) and centrifuged (350 x *g*, 5 min, 4°C). The supernatant was employed to measure the exuded dye colorimetrically (640 nm) and the results were expressed as arbitrary units (a.u.). The pellet was resuspended in PBS (1 ml) to perform total leukocyte counting in a Neubauer chamber of cells stained with Türk solution. Differential counting was performed by staining in hematoxylin, followed by cell fixation in methanol and eosin staining. The percentage of polymorphonuclear and mononuclear cells was obtained considering 100 cells per field.

### Endothelial cell culture

The endothelial cell culture and its characterization followed a method recently described, with slight modifications [20]. Swiss control and infected mice (n = 5 per group) were anesthetized and sacrificed by cervical dislocation and bathed in 70% ethanol. The mesenteric microvasculature was surgically removed in a sterile environment, cut into small pieces and covered with DMEM medium supplemented with 20% heat-inactivated fetal bovine serum, NaHCO_3_ (44 mM), glucose (11 mM) and gentamicin (30 µg/ml) (pH 7.4). The plates were maintained in an incubator (37°C, 5% CO_2_). The explants were removed after 48 h and the medium replaced every 48 h. After confluence, cells were subcultured using the enzyme pancreatin.

The endothelial cell cultures were characterized through morphological analysis (optical microscopy) and with flow cytometry to quantify the surface expression of platelet endothelial cell adhesion molecule (PECAM-1, CD31), a marker of endothelial cells. Briefly, cultured endothelial cells formed a cobblestone monolayer. Primary cultures of endothelial cells were detached by enzymatic digestion with diluted pancreatin and centrifuged (600 x *g*, 20 min) in PBS containing 0.1% sodium azide and 1% bovine serum albumin (FACS buffer). Next, 10^5^ cells were first incubated with Fc blocker (Clone 2.4G2, BD Pharmingen) for 10 min before incubation with rat anti-mouse CD31 biotinylated primary antibodies (BD Pharmingen; 1∶100) or with appropriate fluorochrome-conjugated isotype control antibodies (rat IgG2a, κ isotype control, BD Pharmingen; 1∶100) for 20 min at 4°C. Cells were then washed twice in cold FACS buffer (600 x *g*, 20 min) and incubated with the secondary antibody FITC-conjugated streptoavidin (BD Pharmingen; 1∶100, 20 min at 4°C). After two additional washings (600 x *g*, 20 min), the cells were analyzed using fluorescence-activated cell sorting (FACScalibur, BD). The fluorescence was detected in the fluorescence 1 channel (FL1; 488 nm for excitation and 520 nm for emission, argon-ion laser) and 10,000 events per sample were collected and analyzed using CellQuest Software (BD Pharmingen). Cell gating, forward (FSC) and side (SSC) scatter and fluorescence histograms (FL1) were used for analysis and revealed in both experimental groups a single population of cells that were positive for CD-31 (94–98%) and similar to murine endothelial cells described elsewhere [21].

### Leukocyte-endothelial cell adhesion assay

Fresh blood (approximately 2 ml) was collected from the heart of anesthetized animals, diluted in PBS (1∶1), and carefully layed onto the Ficoll-Paque PLUS (3 ml) following manufactureŕs instructions. After centrifugation (400 x *g*, 30 min, 4°C) the interface containing mainly mononuclear cells was separated (>95% viability) and used for adhesion experiments [22]. Next, first passage mesenteric endothelial cells (10^5^ cells/well) plated 48 h before (96-wells plate) were washed with DMEM and incubated either with vehicle (sterile PBS), tumor necrosis factor (TNF; 0.1 ng/ml, diluted in PBS + BSA 0.1%), TNF plus S-Nitroso-N-Acetyl-DL-Penicillamine (SNAP; 1 µM) or N^G^-nitro-L-Arginine (L-NNA, 300 µM) for 4 h in DMEM (37°C, 5% CO_2_). After this period, the medium was removed and endothelial cells were washed with PBS. The mononuclear cells were added (10^4^/well) and incubated for further 30 min in DMEM (37°C, 5% CO_2_). Non-adherent cells were removed by washing with PBS. Alternatively, a cross-culture model was performed, characterized by the incubation of control endothelial cells with mononuclear leukocytes obtained from infected animals or the opposite, *i.e*., the incubation of endothelial cells obtained from infected mice with control mononuclear leukocytes. We analyzed four fields per well, randomly chosen, and the number of adherent leukocytes in each field was determined using an Olympus IX71 microscope (magnification 400X) equipped with DP2-BSW software (Olympus America Inc, USA).

### NO measurements *in vitro* and immunocytochemistry

Confluent endothelial cells obtained from both experimental groups (first passage; 96-wells plate) were incubated for 45 min with the NO indicator 4-amino-5-methylamino-2′,7′-difluorofluorescein diacetate (DAF-FM DA, 2.5 µM) in the presence of physiological solution (mM: NaCl 140, KCl 5, MgCl_2_ 1, CaCl_2_ 2, glucose 5 and HEPES 5, pH 7.4) enriched with L-arginine (1 mM) (37°C, 5% CO_2_) [20]. NO production was induced by either ATP (100 µM) or the Ca^2+^ ionophore A23187 (2 µM). The probe DAF-FM is very selective for NO [23]. After incubation, the fluorophore trapped in live cells was excited at 488 nm and emission was measured at 515 nm using a microplate fluorometer (Fmax; Molecular Devices, USA). The ATP- and A23187-induced NO production was measured as the difference between the NO content measured in the presence (total) and absence (basal) of the drugs, and expressed as percentage of the basal considered as 100%.

Alternatively, first passage endothelial cells (10^4^ cells/well) were plated on glass coverslips 24 h before immunostaining, put in a 24-well plate and incubated as previously described. In the next day, cells were fixed for 5 min at room temperature in 4% paraformaldehyde diluted in PBS, washed and then incubated for 30 min with ammonium chloride (50 mM, pH 8.0). Following, cells were washed three times with PBS and further incubated for 30 min with a blocking solution (BSA 1% diluted in PBS and non-fat milk, 1∶1). Next, cells were washed three times with Triton 0.2% (diluted in PBS) and incubated overnight (4°C) with polyclonal antibody against eNOS (1∶100, Santa Cruz Biotechnology). Subsequently, cells were washed three times with Triton 0.2% and incubated with biotinylated goat anti-rabbit IgG secondary antibody (1∶300, Vector), followed by three washes. Finally, cells were incubated with Texas Red streptavidin (1∶100, Vector) and then washed again. The coverslips were mounted on slides in the presence of DAPI (Vectashield®, Vector). Analysis of cells was performed by fluorescence microscopy using an Olympus IX71 microscope equipped with DP2-BSW software (Olympus America Inc, USA).

### Western blotting assays

Confluent endothelial cells (first passage) were washed with PBS and 200 µl RIPA buffer was added (1% Nonidet P40, 0.25% sodium deoxycholate, 150 mM NaCl, 1 mM EDTA, 1 mM phenylmethylsulfonyl fluoride (PMSF), 1 mM sodium orthovanadate, 1 mM NaF, 10 µg/ml aprotinin, 10 µg/ml leupeptin, and 50 mM Tris-HCl, pH 7.4). Cell lysates were incubated for 30 min at 4°C and then centrifuged (8,100 x *g*, 10 min, 4°C), and the content of protein was determined using Coomassie Blue dye (BioRad Laboratories). Western blot was performed as previously described [24]. Briefly, 20 µg protein were loaded on SDS-PAGE (7.5%) and, after electrophoresis, proteins were transferred to a nitrocellulose membrane, incubated for 1 h with non-fat milk (5%) followed by primary antibody treatment (polyclonal anti-eNOS, 1∶300, or monoclonal anti-caveolin-1, 1∶3,000, Santa Cruz Biotechnology) and peroxidase-conjugated secondary antibody treatment (anti-rabbit IgG, 1∶500, Santa Cruz Biotechnology). Rouge of Ponceau dye was used as an internal control of protein loading. Protein blot images were scanned by a densitometer (model GS-700, Bio-Rad Laboratories, USA), and the relative optical density was obtained using the Quantity One imaging system software (Bio-Rad Laboratories, USA). Data were normalized and expressed as percentages in relation to controls. Rat brain hemispheres preparation was used as positive control for eNOS antibody (data not shown).

### Data analysis

Data are presented as the arithmetic mean ± S.E.M. of the indicated number of observations. Unpaired Student's *t* test was performed to determine the significance of the differences between two groups. Alternatively, one-way analysis of variance (ANOVA) was used (more than two groups), followed by post hoc Bonferroni's Multiple Comparison test. All tests were performed considering *P*<0.05.

### Drugs

ATP, A23187, L-Arginine, N^G^-nitro-L-Arginine (L-NNA), S-Nitroso-N-Acetyl-DL-Penicillamine (SNAP), tumor necrosis factor (TNF), phenylmethylsulfonyl fluoride (PMSF), sodium orthovanadate, aprotinin and leupeptin were purchased from SIGMA Chemical Co. (St. Louis, MO, USA); DAF-FM DA was obtained from Molecular Probes (Eugene, OR, USA). Ficoll-Paque PLUS was obtained from GE Healthcare (Piscataway, NJ, USA). DMEM, fetal bovine serum and gentamicin reagent solution were acquired from GIBCO BRL Products (Grand Island, NY, USA). Stock solutions were prepared in 100% dimethylsulphoxide (DAF-FM DA 2.5 mM; A23187 5 mM), buffered physiological solution (ATP 10 mM), sterile PBS (LPS 1 mg/ml; SNAP 10 mM), 0.05 M NaOH (L-NNA 10 mM) and diluted daily in buffered physiological solution or sterile PBS. The highest final concentration of the solvent was 0.1% (v/v) and had no effect on the experiments. Antibodies against eNOS, caveolin-1 and secondary antibodies were obtained from Santa Cruz Biotechnology (Santa Cruz, CA, USA). Texas Red streptavidin and biotinylated goat anti-rabbit IgG secondary antibody were obtained from Vector (Burlingame, CA, USA). Antibodies against CD31, isotype control and Fc blocker were obtained from BD Pharmingen (San Jose, CA, USA).

## Results

**Figure 1:**
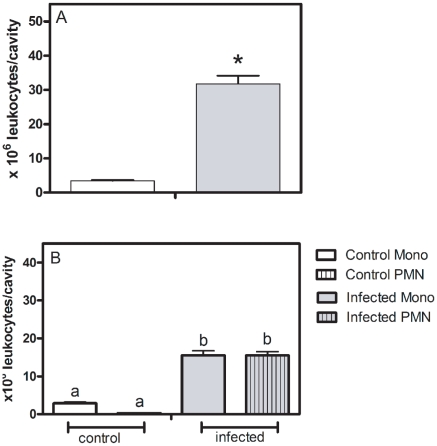
Schistosomiasis increases leukocyte migration into peritoneal cavity *in vivo*. A) Number of infiltrated leukocytes obtained from control (white bar) and *S. mansoni-* infected (gray bar) mice. Data expressed as the mean and S.E.M. *P<0.05, Students *t* test. B) Differential counting of peritoneal infiltrated leukocytes obtained from control (white bars) and *S. mansoni-*infected (gray bars) mice. *P*<0.05 for a vs b, one-way ANOVA followed by Bonferroni's Multiple Comparison test, n = 5–9.

A hallmark of inflammation is leukocyte rolling on the endothelium followed by firm adhesion and infiltration. The total number of cells in the peritoneal exudate was significantly greater in *S. mansoni-*infected than in control mice (Fig. 1A). Differential analysis showed that infection stimulates the migration of both mononuclear and polymorphonuclear cells into the cavity, each one corresponding to 50% of the total number of cells (Fig. 1B). Additionally, measuring peritoneal leakage of intravenously injected Evans blue dye, an index of exudate albumin concentration, it was observed an increased vascular permeability in the peritoneal cavity of infected mice. Protein leakage into the cavity was approximately 2.5 times higher in infected (0.42±0.03 a.u., n = 8, *P*<0.05) than in control animals (0.16±0.02 a.u., n = 8).

Taking into account that we observed an increased leukocyte transmigration to the extravascular space in the infected group, and considering that some reports have shown that endothelial cells primed *in vivo* keep the information in culture [13], [14], we further examined *in vitro* if endothelial cells and leukocytes obtained from infected animals would behave similarly to the *in vivo* condition.

**Figure 2:**
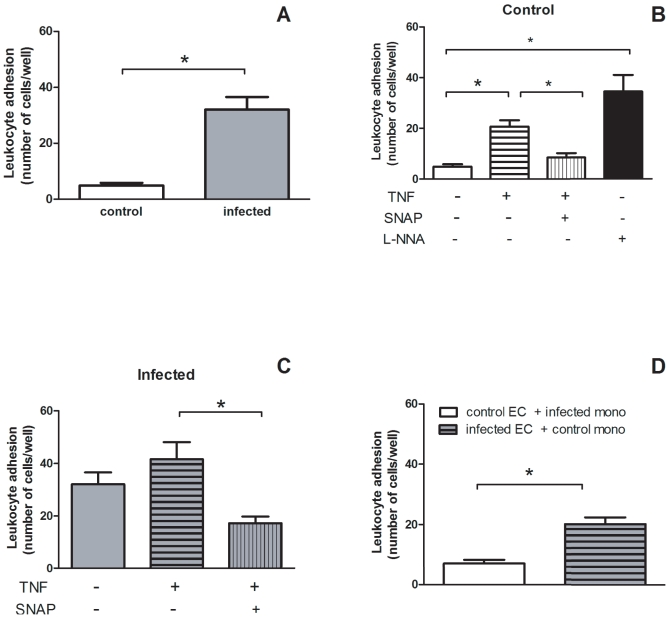
Schistosomiasis increases leukocyte adhesion to mesenteric endothelial cells *in vitro*. Data expressed as the mean and S.E.M. **A.** Number of mononuclear leukocytes adhering to endothelial cell monolayer obtained from control (white bar) and *S. mansoni-*infected mice (gray bar). *P<0.05, Students *t* test. **B.** Number of mononuclear leukocytes adhering to endothelial cell monolayer both from control mice in the absence (open white bar) or presence of TNF (0.1 ng/ml), TNF (0.1 ng/ml) plus SNAP (1 µM) or L-NNA (300 µM) treatment for 4 h. *P<0.05 , One-way ANOVA followed by Bonferroni's Multiple Comparison test. **C.** Number of mononuclear leukocytes adhering to endothelial cell monolayer from *S. mansoni*-infected mice in the absence (open gray bar) or presence of TNF (0.1 ng/ml) or TNF (0.1 ng/ml) plus SNAP (1 µM) treatment for 4 h. *P<0.05, One-way ANOVA followed by Bonferroni's Multiple Comparison test. **D.** Number of mononuclear leukocytes adhering to endothelial cell monolayer. White open bar  =  control endothelial cells (EC) incubated with mononuclear leukocytes (mono) from *S. mansoni*-infected mice. Gray bar with horizontal lines  =  endothelial cells (EC) from infected mice incubated with control mononuclear leukocytes (mono). *P<0.05, Student's *t* test. n = 16 replicates of a typical experiment. TNF  =  tumor necrosis fator; SNAP  =  S-Nitroso-N-Acety-DL-Penicillamine; L-NNA  =  N^G^-nitro-L-Arginine.

Using isolated mononuclear leukocytes and mesenteric endothelial cell cultures obtained from each experimental group (*i.e.,* control and infected mice), the number of adherent leukocytes to endothelial monolayer was about six times higher in infected than in control group (Fig. 2A). As expected, in the control group the treatment with the pro-inflammatory cytokine TNF (0.1 ng/ml, 4 h) increased the number of adherent cells (Fig. 2B). A similar profile was observed in the infected group (Fig. 2C), but the final number of adherent leukocytes to endothelial cells was higher in the infected as compared to control group (Fig. 2B and 2C, 41.6±6.5 and 20.7±2.53 cells/field, respectively, n = 16, *P*<0.05, Students *t* test).

Amongst the endothelium-derived mediators that inhibit mice leukocyte adhesion, nitric oxide (NO) is one of the most studied. Accordingly, co-administration of the NO donor SNAP (1 µM) with TNF reversed the increase of leukocyte adhesion in both experimental groups (Fig. 2B and 2C). However, in the infected group NO donation reduced leukocyte-endothelial cell interaction to a level lower than the basal condition (32.1±4.5 and 17.3±2.53 cells/field, respectively, n = 16, *P*<0.05, Students *t* test), probably reflecting a repair of NO-dependent endothelial cell function (Fig. 2C). In order to confirm the inhibitory effect of endothelial NO on leukocyte adhesion, we performed the inhibition of eNOS from control endothelial cells with L-NNA (300 µM) before adding the control leukocytes. In this condition, leukocyte adhesion in the control group (Fig. 2B) was similar to the one observed in the infected group (Fig. 2A). Furthermore, in a different protocol using a cross-culture model, the incubation of control endothelial cells with leukocytes obtained from infected animals showed values of adhesion similar (Fig. 2D) to the control condition (Fig. 2A), suggesting that the disease affects mostly the endothelial cells. To confirm this hypothesis, we incubated endothelial cells obtained from infected mice with control leukocytes. As expected, an increased leukocyte adhesion was observed (Fig. 2D), mimicking somehow the result of the infected group (Fig. 2A). Consequently, the next step was to investigate NO production by cultured endothelial cells from infected animals.

**Figure 3:**
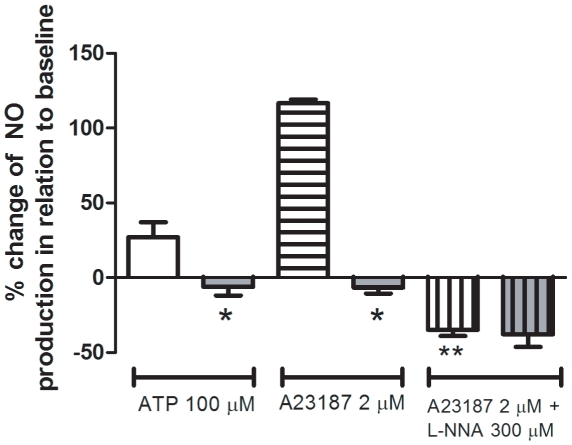
Nitric oxide production in cultured endothelial cells from control and *S. mansoni*-infected mice. Nitric oxide (NO) production by confluent mesenteric endothelial cells in response to 100 µM ATP and 2 µM A23187 was measured in living cells using the fluorescent probe DAF-FM (2.5 µM) and a microplate fluorometer. Control mice: white bars (open, horizontal and vertical lines). Infected mice: gray bars (open, horizontal and vertical lines). Data are expressed as the mean and S.E.M. of 6–7 experiments performed in triplicate obtained from four different cultures and from different animals (ATP condition) or four replicates of a typical experiment (A23187 condition). Basal NO production observed in the absence of ATP or A23187 was considered as 100%. *P<0.05 vs. control mice; **P<0.05 vs. A23187 treatment in control mice, One-way ANOVA followed by Bonferroni's Multiple Comparison test.

**Figure 4:**
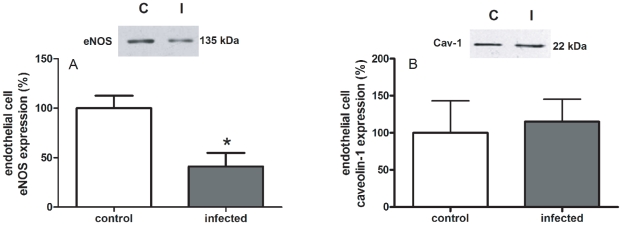
Expression of eNOS and caveolin-1 in endothelial cells from control and *S. mansoni*-infected mice. C  =  control; I  =  infected. A refers to eNOS expression; B refers to caveolin-1 (Cav-1) expression in confluent cultured mesenteric endothelial cells. *Insert*: Representative experiment of each experimental group. Rouge of Ponceau dye was used as an internal control of protein loading and did not differ among samples (data not shown). Data expressed as the mean and S.E.M. *P = 0.014 (Students *t* test), n = 3 (caveolin-1) or 5 (eNOS).

**Figure 5:**
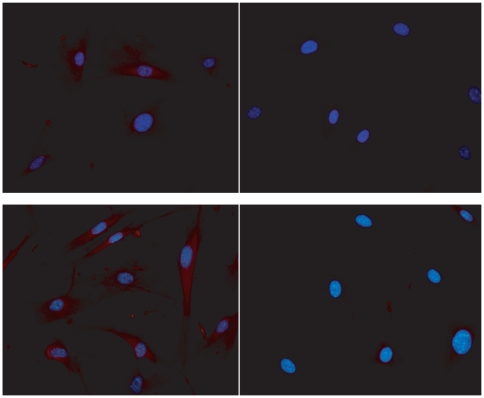
Expression of eNOS and caveolin-1 in endothelial cells from control and *S. mansoni*-infected mice. Merged images of the immunocytochemical staining of cultured endothelial cells using an antibody against eNOS (red) and nuclear fluorescence using DAPI (blue) (400X). Upper and lower left panels: controls. Upper and lower right panels: *S. mansoni*-infected mice. Each image from control and infected groups was randomly chosen and obtained from a different plate.

In CD-31 positive cultured endothelial cells obtained from mesenteric vessels, we investigated NO production in response to ATP, a classic agonist of purinergic P2 receptors that activates eNOS [20] or A23187, a Ca^2+^ ionophore that also activates eNOS but in a receptor-independent way. NO production in response to 100 µM ATP increased approximately 27% over basal condition in the control group, which is compatible with the literature [20]. However, such increase in NO production was not observed in the infected group either using ATP or A23187 (2 µM) (Fig. 3). The inhibition of eNOS with L-NNA (300 µM) fully prevented A23187 effect in the control group confirming the specificity of the measurement (Fig. 3). In order to discriminate whether the absence of ATP- and A23187-induced production of NO in the infected group was due to a reduced activity or expression of the enzyme, we evaluated the expressions of eNOS and of caveolin-1, a known inhibitor of eNOS through protein-protein interaction. Data obtained showed a single band corresponding to the molecular weight range of 22 kDa and another band in 135 kDa being compatible with caveolin-1 and eNOS, respectively (Fig. 4). Schistosomiasis down-regulated eNOS expression as quantified by western blotting assays (Fig. 4A). Similar qualitative results were observed by immunocytochemistry (Fig. 5). However, schistosomiasis did not alter the expression of caveolin-1 (Fig. 4B).

## Discussion

Endothelial cells constitute the inner cellular lining of blood vessels being an interface between blood and vascular tissue, and regulate many physiological functions including vascular tonus, inflammation and immunity [10], [11]. They are involved in most disease states either as a determinant of the pathophysiology or as target of a collateral injury. Despite the fact that schistosomiasis is an intravascular parasitic disease its possible impact on endothelial cell function has been poorly explored either *in vivo* or *in vitro*. The present study provides evidence supported by *in vivo* and *in vitro* assays that schistosomiasis increases endothelial cell-leukocyte interaction and vascular permeability. Such events are related to a reduced expression of eNOS, a key endothelial cell enzyme.

The vascular endothelium, which plays an integral role in the regional specialization of vascular structures, is a highly heterogeneous tissue due to differences in the extracellular environment [25]–[28]. Schistosomiasis is characterized by the production of a repertoire of Th_1_ and Th_2_ host cytokines [5], and some of them are well known for their injurious effects on endothelial cell function [11].

The increased number of infiltrated leukocytes and protein concentration in the peritoneal cavity of the infected animals show that they present an inflamed cellular and vascular profile. We also observed by intravital microscopy an increase of spontaneous leukocyte rolling in mice cremaster microcirculation in infected animals (unpublished results).

Endothelial cells keep in culture the phenotype of the time in which they were removed from the donors, *i.e*., the result of epigenetic effects may be maintained in long-term cultured cells [28], as shown either in cells obtained from rats [13], [14] or humans [28]–[30]. Taking into account this possibility, we performed *in vitro* assays using cultured endothelial cells and mononuclear leukocytes obtained from control or *S. mansoni*-infected mice to further investigate the influence of the disease on leukocyte adhesion.

The quantification of leukocyte adhesion to cultured mesenteric endothelial cells revealed an increased number of adherent leukocytes in the infected group, either in basal or TNF-treated conditions, being the later fully prevented by NO donation. The number of basal adherent leukocytes to endothelial cells was about six times higher in infected than in control mice. Since in the infected group NO donation reduced leukocyte-endothelial cell interaction to a level lower than the basal condition this could reflect a repair of NO-dependent endothelial cell function. Accordingly, in the control group eNOS inhibition induced a leukocyte adhesion level similar to the one observed in the infected group, corroborating the inhibitory effect of NO on leukocyte adhesion. Noteworthy, leukocytes from control mice adhered to endothelial cells from infected mice in a similar way that was observed in the infected group, confirming that schistosomiasis alters endothelial cell function.

Endothelial cells are a major determinant of leukocyte adhesion and vascular permeability, and under normal conditions, they provide a well-known anti-inflammatory state [9]. To achieve these functions, gene expression patterns are tightly regulated in endothelial cells. In this regard, eNOS expression plays an important role. For instance, in eNOS-null mice, but not in inducible NOS-null mice, there is a significant increase in the number of rolling leukocytes [31], [32] and vascular permeability [7] suggestive of an up-regulation of inflammatory reaction in conditions of reduced eNOS-derived NO [33]. In support to this idea, inhibition of eNOS increases leukocyte adhesion ([34] and present work) and migration in the murine peritoneal cavity [35], leukocyte adhesion to mesenteric vascular endothelium [31] and vascular permeability [7]. Therefore, we might suppose that eNOS-derived NO suppress endothelial cell activation in an autocrine fashion counteracting signals that mediate their activation. For instance, NO inhibits the endothelial expression of adhesion molecules such as intercellular adhesion molecule (ICAM)-1, which is essential for endothelial cell-leukocyte interaction [36]. In this context, in schistosomiasis, there is an increase in the expression of ICAM-1 [37] and plasma soluble ICAM-1 [12], a marker of inflammatory diseases and endothelial activation.

Vascular endothelial cells are the primary eNOS-expressing cell type being eNOS mRNA constitutively expressed. Steady-state eNOS mRNA levels are regulated at epigenetic, transcriptional and post-transcriptional levels [33], [38]. The knowledge about the mechanism and role of epigenetic regulation of eNOS expression continues to mount [38]. In addition, the enzymatic activity is subject to post-translational regulation through protein-protein interactions. One such negative regulator is caveolin-1 [39], [40].

Previous indirect data suggested a reduced production of NO in mice portal vein [1], but considering the phenotypic heterogeneity of endothelial cells [26], [28], it was necessary to investigate NO production and eNOS expression in the present model.

In fact, an impairment of ATP-induced NO production in cultured endothelial cells from infected animals was found in the present work, whereas NO production in the control group was similar to the level previously reported in similar experimental conditions [20]. As a similar result was observed with A23187, which activates eNOS in a receptor-independent manner, we may disregard the hypothesis that the lack of effect of ATP in the infected group is due only to a defect of purinergic signaling. As recently proposed, the innate immune response reduces endothelial NO production [41]. Furthermore comparing the eNOS expression in both groups we observed a reduction in mesenteric endothelial cells from infected compared to control mice, with no alteration of caveolin-1 expression, a known repressor of eNOS activity. Finally, the number of basal adherent leukocytes to mesenteric endothelial cells in our model was similar to previous data in mesenteric vessels of eNOS-deficient mice [31]. All evidence of the current model reinforce the important role of eNOS to the integrity of the microcirculatory endothelial barrier *in vivo*, as observed in other experimental models [7], [35]. Most probably there may be other phenotypic alterations of endothelial cells not addressed in this work that also contribute to the increased leukocyte adhesion. Therefore, firstly we propose that the increased endothelial cell-leukocyte interaction, and probably vascular permeability, in murine schistosomiasis are partially related to the reduced eNOS expression. Additionally, the mesenteric endothelial cells of infected mice keep in culture the phenotypic profile of their donor animal as described elsewhere [13], [14], [29]–[30]. Consequently, it is herein suggested that schistosomiasis primes murine endothelial cells so that they keep the information of increasing leukocyte adhesion in culture, making endothelial cells culture a putative model to study *in vitro* the consequences of the disease.

The mediator involved in the reduction of eNOS expression in this model has not been identified so far, and is beyond the scope of the present study. However, the disease provides a repertoire of modulators for the host immune system. Hence, it is reasonable to suppose that the reduced expression of eNOS may reflect the balance of the effects of parasite-derived molecules and host cytokines, *i.e.,* an integrated network of biological events, rather than the effect of a single mediator.

In summary, our data show that murine schistosomiasis increases vascular permeability and leukocyte-endothelial interaction while reduces the expression of eNOS, promoting an inflamed cellular profile in peritoneum. Additionally, mesenteric endothelial cells from *S. mansoni*-infected mice suffer a phenotypic change that is maintained in culture suggesting that the disease probably triggers epigenetic regulation of endothelial cells.
